# miRiaD: A Text Mining Tool for Detecting Associations of microRNAs with Diseases

**DOI:** 10.1186/s13326-015-0044-y

**Published:** 2016-04-29

**Authors:** Samir Gupta, Karen E. Ross, Catalina O. Tudor, Cathy H. Wu, Carl J. Schmidt, K. Vijay-Shanker

**Affiliations:** Department of Computer and Information Sciences, University of Delaware, Newark, DE 19711 USA; Center for Bioinformatics and Computational Biology, University of Delaware, Newark, DE 19711 USA; Department of Food and Animal Sciences, University of Delaware, Newark, DE 19711 USA

**Keywords:** MicroRNA, Disease, Associations, Text-mining, Relation extraction, Natural language processing

## Abstract

**Background:**

MicroRNAs are increasingly being appreciated as critical players in human diseases, and questions concerning the role of microRNAs arise in many areas of biomedical research. There are several manually curated databases of microRNA-disease associations gathered from the biomedical literature; however, it is difficult for curators of these databases to keep up with the explosion of publications in the microRNA-disease field. Moreover, automated literature mining tools that assist manual curation of microRNA-disease associations currently capture only one microRNA property (expression) in the context of one disease (cancer). Thus, there is a clear need to develop more sophisticated automated literature mining tools that capture a variety of microRNA properties and relations in the context of multiple diseases to provide researchers with fast access to the most recent published information and to streamline and accelerate manual curation.

**Methods:**

We have developed **miRiaD** (**mi**cro**R**NAs **i**n **a**ssociation with **D**isease), a text-mining tool that automatically extracts associations between microRNAs and diseases from the literature. These associations are often not directly linked, and the intermediate relations are often highly informative for the biomedical researcher. Thus, miRiaD extracts the miR-disease pairs together with an explanation for their association. We also developed a procedure that assigns scores to sentences, marking their informativeness, based on the microRNA-disease relation observed within the sentence.

**Results:**

miRiaD was applied to the entire Medline corpus, identifying 8301 PMIDs with miR-disease associations. These abstracts and the miR-disease associations are available for browsing at http://biotm.cis.udel.edu/miRiaD. We evaluated the recall and precision of miRiaD with respect to information of high interest to public microRNA-disease database curators (expression and target gene associations), obtaining a recall of 88.46–90.78. When we expanded the evaluation to include sentences with a wide range of microRNA-disease information that may be of interest to biomedical researchers, miRiaD also performed very well with a F-score of 89.4. The informativeness ranking of sentences was evaluated in terms of nDCG (0.977) and correlation metrics (0.678-0.727) when compared to an annotator’s ranked list.

**Conclusions:**

miRiaD, a high performance system that can capture a wide variety of microRNA-disease related information, extends beyond the scope of existing microRNA-disease resources. It can be incorporated into manual curation pipelines and serve as a resource for biomedical researchers interested in the role of microRNAs in disease. In our ongoing work we are developing an improved miRiaD web interface that will facilitate complex queries about microRNA-disease relationships, such as “In what diseases does microRNA regulation of apoptosis play a role?” or “Is there overlap in the sets of genes targeted by microRNAs in different types of dementia?”.”

**Electronic supplementary material:**

The online version of this article (doi:10.1186/s13326-015-0044-y) contains supplementary material, which is available to authorized users.

## Background

MicroRNAs (miRs) are a class of small non-coding RNAs encoded in the genomes of animals, plants, and protozoa. In general, miRs negatively regulate gene expression by base pairing with sequences in the 3’-untranslated region of mRNAs, which either inhibits their translation or triggers their cleavage. Thousands of miRs have been identified in mammals, and they have been implicated in the control of a wide range of biological processes [1].

miRs are increasingly being appreciated as critical players in human disease. The role of miRs in cancer is very well established, with a wealth of studies demonstrating the participation of miRs in multiple cancer-related processes in diverse tissue types [2]. miRs have also been linked to many other diseases, including cardiovascular disease [3], diabetes [4], neurological disease [5] and liver [6] and intestinal [7] disorders.

Although at a mechanistic level miRs influence disease through their effects on the expression of their target genes, in the scientific literature miRs are associated with diseases through a variety of relationships. In some cases, miRs are directly associated with the disease itself or with a feature or outcome of the disease, such as aggressiveness [8], invasiveness [9], or patient survival [10]. In other cases, miRs are identified as biomarkers [11] or therapeutic targets [12] for a disease. miRs can also be linked to biological processes that are, in turn, connected to the disease. This category includes miR-gene targeting events, as well as regulation of processes such apoptosis [13], metastasis [14], or cholesterol transport [15] by miRs. Finally, in some cases, it is the state of the miR (e.g., over- or under-expression [16]) that is associated with the disease.

There are currently several high-quality databases that capture miR-disease associations and some of the above relations, including miR2Disease [17], miRCancer [18] and the Human microRNA Disease Database (HMDD; [19]). These resources are literature based and support searches for miR or disease of interest. miR2Disease and miRCancer provide information on miR expression in disease, and miR2Disease additionally covers miR target genes. HMDD documents miRs that are potential biomarkers and provides several analysis tools, such as miR set enrichment analysis. miR2Disease and HMDD are manually curated; thus they are limited by the time-consuming nature of manual curation and have difficulty keeping up with the explosion of publications in the miR-disease field. In 2014 alone, using the PubMed query “*miRNA*[*TIAB*] *OR microRNA*[*TIAB*] *OR miR*[*TIAB*]”, we obtained around 5100 PubMed citations, which was a 120 % increase compared to 2013 and a 160 % increase compared to 2012. Additionally, we identified 19,402 abstracts (as of February 2015) that mention miRs and of these, we estimate 15,171 abstracts also mention disease terms (as detected by PubTator [20]).

Automated literature mining tools could help streamline and accelerate the curation process as well as provide researchers with fast access to the most recent published information; however, currently, such tools are limited and have not been widely adopted. Most of the miR-related literature mining tools available focus on extraction of miR-target gene relations without regard to disease, and rely on relatively simple text mining techniques, such as co-occurrence of miR and disease in the same sentence or abstract. These include miRSel [21] and the tools used by the miR-target databases miRWalk [22], TarBase [23], and miRTarBase [24]. miRCancer [18], is one of the few resources that uses a rule-based system to identify disease-relevant miR information in literature, but is limited to detecting miR expression associations in cancer.

In this work, we present **miRiaD** (**mi**cro**R**NAs **i**n **a**ssociation with **D**isease), which automatically extracts from the biomedical literature associations between miRs and diseases together with any intermediate relations that bridge the association, thereby capturing “myriad” ways in which a miR can be associated with a disease. In general miRiaD connects a miR or an “aspect” of a miR (e.g., differential expression, methylated state) to a disease or a disease “aspect” (e.g., outcome or therapy) through some relations (e.g., involvement, regulation, is-a). For example, miRiaD can extract a miR’s involvement in the outcome of a disease, or its role as a biomarker or therapeutic target for a disease. Additionally, miRiaD can extract the involvement of a miR in some cellular process that is highly related to a disease, thus (indirectly) linking the miR with the disease. These links between a miR and a disease through cellular processes or target gene, which we refer to as “linking entity”, are often implicit but highly informative to researchers studying disease mechanisms. Our previous work, STEM [25], extracted relations between two entities, namely a miR and process/function terms. miRiaD extends upon the previous work by allowing more type of entities to be linked (e.g. disease with its outcome, miR with a disease outcome etc.).

We have applied miRiaD to the entire set of Medline abstracts, and we provide a web interface through which the results can be searched using PubMed-like queries. Details about our miR-disease association extraction approach are presented in the Methods section; screenshots and details about the interface are provided in the Results and Discussion section. In conjunction with miRiaD, we developed a procedure for ranking sentences containing miR and disease mentions according to their “informativeness,” which we envision can be used in the future to guide how miRiaD results will be presented to the user. The details of this approach are given in the Methods section.

miRiaD was evaluated with two potential user communities—miR-disease database curators and biomedical researchers--in mind. To address the needs of curators, the recall and precision of miRiaD was evaluated with respect miR-target and miR expression information, which are the two types of information curated by miR2Disease, the most comprehensive database for miR-disease associations; this evaluations achieved recall results between 88.46–90.78 %. For biomedical researchers, who are potentially interested in the full range of possible connections between miRs and disease, we evaluated miRiaD with respect to a variety of sentences in which miRs and diseases co-occur, resulting in F-scores of 89.4 %. Finally, an evaluation of our informativeness ranking system accomplished an nDCG of 0.9815, as well as correlations of 0.678–0.727, when compared to an annotator’s ranked list. Details about the experimental setup and the evaluations are given in the Results and Discussion section.

## Methods: Approach and Implementation

**Fig. 1:**
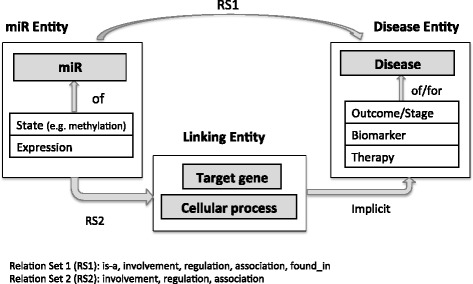
miR-disease associations extracted by miRiaD. miRs or their aspects (state or expression levels) can be directly associated with diseases or disease aspects (outcome, stage, biomarker/therapy) through a variety of relations; association can also be bridged by a linking entity such as a target gene or biological process

In developing the miRiaD system, we attempted to capture the variety of ways in which connections between a miR and a disease are stated in text. Figure 1 schematically depicts these relationships. First, both miRs and diseases are often associated with descriptive information or properties, which we will collectively refer to as “aspects.” Examples of miR aspects include expression level and state (e.g., hyper-methylated or mutated); examples of disease aspects include outcome/stage, biomarker, or therapy. For convenience, we will refer to a miR or its aspects as a miR *entity* (e.g. mir-9, overexpressed mir-9, hypermethylation of mir-9) and likewise refer to a disease or its aspects as a *disease entity* (e.g. gastric cancer, biomarker for gastric cancer). In some sentences, a miR entity may be directly related to a disease entity. In other cases, a miR entity may regulate a target gene or be involved in a biological process that is in turn implicitly linked to a disease entity. Even more complex associations are possible; for example, a miR may regulate a gene that is involved in a biological process that is ultimately relevant to a disease. These relationships may be expressed in text using a variety of phrases and not all phrases are applicable to all types of relationships. An association between a miR and a process or disease is likely to be described using relations such as “involved in” or “has a role in”, whereas an association between a miR and biomarker is likely to be expressed using an “is-a” relation. A formal description of the patterns, the list of triggers and the types relations between the different pairs is provided in Additional file 1.

miRiaD identifies specific relations (i) between a miR and its aspect, (ii) between a disease and its aspect and (iii) between a miR entity and a disease entity. For example, in the sentence “Downregulation of **mir**-**26a** is associated with tumor metastasis in **osteosarcoma**.”, miRiaD will detect the connection between mir-26a and its aspect, downregulation; between the disease osteosarcoma and its aspect, tumor metastasis; and between miR entity “miR-26a downregulation” and the disease entity of “tumor metastasis in osteosarcoma”.

miRiaD also detects multi-step connections where the connection between the miR entity and disease entity is mediated through another entity or process. We call this extra entity or process *a linking entity*. Consider the sentence, “MicroRNA-9 promotes tumor metastasis via repressing E-cadherin in esophageal squamous cell carcinoma.” Typically, as in this sentence, the miR entity regulates or is involved with the linking entity (E-Cadherin). This regulation in turn can be connected to the disease entity (tumor metastasis of esophageal squamous cell carcinoma). However, it is quite common for the connection between the linking entity and the disease entity to be left unstated with an understanding of the implicit connection requiring additional domain knowledge.

**Fig. 2:**
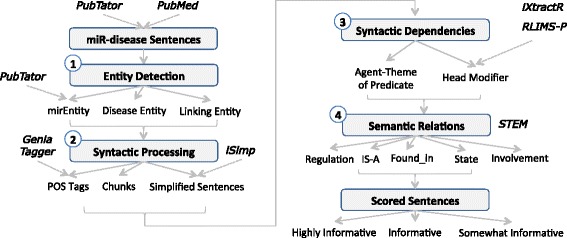
miRiaD Pipeline. The steps of the miRiaD pipeline are illustrated with numbered grey blocks. External tools used throughout the pipeline are shown in bold and italic font

miRiaD currently extracts information from Medline abstracts. After the abstracts are retrieved the abstract text and title are extracted. The text is split into individual sentences using a tool developed in-house. miRiaD extracts the connection between a miR and disease, through the detection of the direct relations between miR entities and disease entities, or through detection of multiple relations involving linking entities. miRiaD uses the presence of certain lexico-syntactic dependency structures in a sentence to detect these semantic relations. Thus, the basic steps of the miRiaD system include (i) Detecting miR/disease entities and “linking entities”; (ii) preliminary syntactic processing; (iii) identifying syntactic dependencies between miRs and co-occurring terms; and (iv) assigning semantic relations between miRs and co-occurring terms. These steps are described below and also shown in Fig. 2. Finally, we also describe a method to score sentences based on their informativeness in describing miR-disease connections.

### Detecting miR/disease entities and linking entities

#### miR entity

A miR entity can be a miR in isolation or together with one of its aspects (expression, mutation, methylation). Although miRs are mentioned in text in a variety of ways (e.g., miR-1, microRNA1, miRNA-1, let-1, etc.), they follow a well-established naming convention. miR mentions consists of a prefix (“miR”, “MIR, “miRNA”, “microRNA”) followed by a unique identifying number, which is assigned based on sequence similarity. This number may be followed by a suffix such as “-a”, “-1”, “-3p” or “-5p”, and/or a prefix that denotes the species may be included. miRiaD detects such miR mentions by using simple regular expressions.

miR aspects usually describe the abundance or properties of a miR. We detect the former by searching for noun phrases headed by trigger words such as “level”, “expression” and “regulation” as well as their variants. For the latter, we consider mutation terms such as “mutation”, “variants” or “polymorphism” as well as nominalized forms of common events such as “methylation”. Of course these terms are only candidates to be miR aspects and are treated as such only after we detect their syntactic relation to a miR.

#### Disease entity

A disease entity can be disease in isolation or in combination with one of its aspects (diagnostic, treatment, outcome). We detect disease mentions using Pubtator [20] database, which includes disease mentions tagged in Medline abstracts by DNorm [26]. The disease mentions are normalized to Medic concept IDs. We process only those abstracts which have a disease mention and thus recall for disease mention detection is important for the miRiaD system performance. The DNorm [26] system reports a micro-averaged precision, recall and f-measure of 0.803, 0.763 and 0.762.

We did some additional analysis for PubTator disease detection by randomly selecting 200 abstracts from the miR2Disease database and checking whether the disease annotated by the miR2Disease curators was detected by Pubtator. A miR2Disease abstract can be annotated with a disease which is not mentioned in the abstract but mentioned in the full length article. Thus we selected only those miR2Disease abstracts where the annotated disease either was mentioned in the title or the abstract. While checking if the annotated disease matched one of the disease mentions detected by Pubtator, we allowed for synonym matches (breast cancer with breast carcinoma). Pubtator picked the exact name, a synonym or part of the annotated disease name in 100 % of the abstracts. However in ten cases, it picked only part of the name, despite the fact that there was ample evidence of the full name mention. For example, in a sentence that contained the phrase “Oral Squamous Cell Carcinoma (OSCC) ”, PubTator only detected Squamous Cell Carcinoma.

Similar to miR aspect detection, we locate disease aspects by looking for certain trigger words or and their textual variations. Commonly occurring disease aspect terms include disease stage or “outcome” terms/phrases like “clinical outcome”, “disease free survival (dfs)”, “overall survival (os)”, “metastasis”, “sensitivity”, “prognosis”, “tumorgenicity”, “invasion”, and “progression”. Diagnosis-related terms include “biomarker”, “marker”, “predictor”, “profiler”, “prognostic”, “diagnosis”, “indicator”, and their textual variations; and finally treatment-related triggers including “therapeutic”, “treatment”, “target”, “therapy”, and their textual variations. As with miR aspects, a candidate phrase is considered to be a disease aspect only after the syntactic dependency (as described later) with a disease mention is established. Several examples of miR entities (“let-7i” and “low miR-335 levels”) and disease entities (“colorectal cancer metastasis”, “overall survival”, and “relapse-free survival”) are highlighted in the following sentence fragments: “**let**-**7i** is associated with **colorectal cancer metastasis**” and “**low miR**-**335 levels** in EOC were associated with shorter **overall survival** and **relapse**-**free survival**”

#### Linking entity

Linking entities are cellular processes or target genes through which the miRs association with the disease can be explained. We use PubTator, which uses GenNorm [27] to identify gene mentions in a sentence. In order to detect cellular processes, we follow the method adopted in eGIFT [28], which uses dictionary look-ups or morphological derivatives e.g., terms/phrases with -sion, −tion, −sis, −or, −er, −ment suffixes. Candidate linking entity phrases are excluded if they are determined to be a miR/disease aspect phrase. Additionally syntactic dependency (as discussed later in this section) needs to be established for the candidate phrase to be considered as a linking phrase. In the sentence fragment “mir-320a down-regulation mediates bladder carcinoma invasion by targeting **itgb3** the linking entity is highlighted.

### Preliminary Syntactic Processing

The miRiaD approach attempts to identify a relation between a miR entity and a disease entity or linking entity by first identifying syntactic dependencies between phrases of that sentence. Two steps facilitate the detection of such syntactic dependencies: chunking and simplification. Note, to reduce overhead for the chunking and simplification step, we filter out sentences that do not contain a miR or a disease mention. Chunking is the task of identifying and grouping words in a sentence into constituents (noun groups, verb groups etc.) called “chunks”. Sentences are tagged with part-of-speech (POS) tags using the Genia Tagger [29]. We further chunk the words based on syntactically related POS tags to form noun phrases (NPs), verb groups (VGs) and prepositional phrases (PPs).

After chunking, we use iSimp [30], which simplifies a variety of complex syntax structures in a sentence into a relatively small number of simple patterns, thus facilitating the identification of syntactic dependencies and relation extraction. iSimp [30] identifies syntactic constructs, such as appositives, relative and reduced relative clauses, conjunctions, and parenthetical elements. These syntactic constructs are used to form simple sentences from a complex sentence. For example, consider the following sentence:

We have profiled four miRNAs, miR-21, miR-210, miR-155, and miR-196a, all implicated in the development of pancreatic cancer with either proven or predicted target genes involved in critical cancer-associated cellular pathways. (PMID 19,723,895)

We illustrate here how miRiaD identifies the relation between miR-21 and the disease entity “pancreatic cancer development”. Automatically detecting a relationship between miR-21 and pancreatic cancer in this sentence might be difficult by just trying to match basic patterns and rules. Additional information of no immediate use occurs between the two mentions, preventing the patterns from detecting a relationship. However, by using simplification constructs identified by iSimp, we can generate a simple sentence from the original sentence that states the relationship in a straightforward way: “Mir-21 is implicated in the development of pancreatic cancer.” iSimp tags the following syntactic constructs:

(i) A conjunction in the form of a list of elements (“miR-21, miR-210, miR-155, and miR-196a”),
(ii)  A conjunction (“proven or predicted”),
(iii)  The appositive construct involving the two noun phrases “four miRNAs” and “miR-21, miR-210, miR-155, and miR-196a”,
(iv)  The reduced relative clause “all implicated in the development of pancreatic cancer with either proven or predicted target genes”, which modifies the noun phrase “four miRNAs”
(v) Another reduced relative clause “involved in critical cancer-associated cellular pathways”, which modifies the noun phrase “target genes”

Using constructs (i) and (iii) we can replace “four miRNAs” by “miR-21” in the construct (iv) and thus generate “miR-21 is implicated in the development of pancreatic cancer …”. Additional simplifications are also generated for the remaining elements of the list (miR-210, miR-155, or miR-196a). Note that the chunking information is carried through when generating simplified sentences from iSimp constructs.

### Identifying Syntactic Dependencies

miRiaD extends our previous system [25], which was built to detect semantic relations, such “involvement”, “regulation”, “state”, “attribute” and “association”, between miRs and biological processes or functional aspects of miRs.. Like STEM, to capture the above semantic relations we detect these semantic relations via the detection of the following lexico-syntactic dependencies. 1) agent-theme of predicate (e.g., “**miR**-**9***regulates***cell proliferation**”), and 2) noun modification (e.g., “**miR**-**1***overexpression*”, “*metastasis* of **gastric cancer**”). We detect these lexico-syntactic dependencies after chunking and sentence simplification.

#### Agent-theme of predicate

We are interested in extracting the relations of “involvement” and “regulation” where a miR entity is the agent and a process (for example) is the theme. These often have a syntactic dependency counterpart (subject and object of a verb). Consider the example sentence “**miR**-**9***regulates***cell proliferation**”. Here the “regulation” relation is represented by the verb “regulate” and the agent (mir-9) and theme (cell proliferation) are the subject and object of this verb. Typically the predicates are simple verbs, and the arguments are noun phrases appearing to the left and to the right of the predicate if the clause is in active voice. However, with the use of chunking, it is possible to extract relationships from more complex sentences as well. Consider the sentence “mir-9 is known to directly regulate cell proliferation”. Notice by chunking this sentence we will get two verb groups (“is known” and “to directly regulate”). The predicate verb (“regulate”) is the head verb of the second of the two consecutive verb groups and thus the noun phrase before this predicate is still “miR-9”. In the sentence “expression of mir-9 regulates proliferation of U87 cells.” the agent and the theme are not base noun phrases, but are larger noun phrases that have attached prepositional phrases, which are identified by chunking.

The voice of the predicate verb is important in determining the role of the arguments. For example, in the sentence “**apoptosis** is *regulated* by **miR**-**9**”, “miR-9” is the agent performing the action “regulates” and “apoptosis” is the theme being regulated. This is the reason why, rather than using syntactic notions of subject and object, we refer to more thematic notions of agent and theme of a predicate, abstracting away from word order.

There are also cases in which the predicate is expressed in a nominalized form. For example, consider the fragment “**miR**-**9***regulation***of cell proliferation**”, where the predicate is the nominalized form of the verb “regulates”. We extend our agent-theme extraction rules to handle such cases by following the rules for nominalization from iXtractR [31].

Predicates need not necessarily be single words. In the sentence “**miR**-**146a** may *play a role in* cell proliferation”, the predicate spans multiple words. Phrases like, “is crucial for”, “is important for”, “plays a role in” are some examples of multi-word predicates that we consider. We defer to the next part of this section for a discussion of the words and phrases that constitute the predicates we are interested in.

In some sentences, an argument (agent/theme) can be shared by two predicates. For example, consider the sentence “**miR**-**126***was able to inhibit***laryngeal squamous cell carcinoma** partly by *suppressing***Camsap1 expression**”. Here, “miR-126” is the agent for both “inhibit” and “suppressing” predicates. Because it is awkward to mention the agent each time one of its predicates is used, the agent is mentioned only once, in the beginning, and omitted in the second case (i.e., for the verb suppress). We call the cases in which the argument is omitted and inferred from an earlier mention the “null-argument” agent-theme of predicate. Sentences with null-arguments have clauses separated by prepositions “to” + a simple verb, “via” or “through” + nominal form of a verb, or “by” + a verb ending in “ing”. For example, “**Tumor suppressive miR**-**1***induces***apoptosis** through *direct inhibition* of **SRSF9** in **bladder cancer**” is a sentence containing a null-argument after “through” + nominal form of a verb. The null argument rules that identify the agent for the second predicate were taken from RLIMS-P [32] and iXtractR [31].

Our rules are limited to some simple patterns corresponding to simple syntactic structures in order to be precise. Sentence simplification allows us to handle more complex cases without making the patterns more involved. Consider the sentence “breast cancer metastasis suppressor 1 up-regulates **mir**-**146**, which suppresses **breast cancer** metastasis”, which contains a relative clause. Without simplification our rules would incorrectly extract [which (agent), suppresses (predicate), breast cancer metastasis (theme)]. iSimp detects the relative clause and generates a simplified sentence “mir-146 suppresses breast cancer metastasis”, thus enabling us to extract the correct relation [miR-146 (agent), suppresses (predicate), breast cancer (theme)]. As discussed in the pre-processing step the other simplification constructs which iSimp handles include appositives, conjunctions, reduced relative clauses and parentheticals.

#### Noun modification

We detect “state” or “attribute” relations that are in a noun modification syntactic relationship. Consider the subject noun phrase in the sentence “**mir**-**21***overexpression* is associated with **glioblastoma**”. The entity “miR-21” acts a modifier of a noun, “overexpression”, which is a nominalized form of a stative verb. In this case the head modifier relation indicates the state of entity “miR-9”. Additionally the head modifier dependency can be stated with a prepositional attachment (e.g., *metastasis* of **gastric cancer**). In this work, we only consider these two forms of noun modification syntactic dependency.

### Assigning Semantic Relations

Having captured the syntactic dependency structures, we look at the predicate for the identification of the semantic relation between a miR entity and a disease entity or a linking entity. In our previous work we observed that the semantic relations that frequently connect a miR entity with a related term (disease entity or linking entity) fall into several categories, namely “involvement”, “regulation”, “is-a”, “association” “found-in” and “state”. Each category encompasses a number of trigger words that are commonly found in text. The predicate detected in the pattern used to detect the syntactic dependency can be used to assign the relation to the appropriate category. For example, if the predicate found in the sentence is “is involved” or “is implicated”, then the relation is categorized as an “involvement” relation. As noted earlier, the verb group containing the predicate can contain additional words modifying the predicate (e.g., “directly targets” or “was able to inhibit”). In these situations, the head of the final verb group is used when matching the trigger words (e.g., “targets” or “inhibit”). Example trigger words and sentences are given below for each of the four categories. Although trigger words are provided in present tense in the examples below, the reader should assume all of their textual variations (tense and nominalized forms).

#### Involvement relations

For detection of the “involvement” relation, we expect the use of the agent-predicate-theme dependency structure. Further, we expect the miR entity and the disease entity or a linking entity to be picked as the agent and theme respectively. A range of words or multi-word triggers can be used as the predicate including: is involved in, is implicated in, is required for, is needed for, play a role in, is necessary/sufficient/crucial/etc. for, is dependent on, participates in, contributes to, influences, fosters, affects, allows, initiates, etc. The main verbs in these triggers can appear in different tense forms and the verb or noun can be modified, as in the sentence “miR-21 may play a critical role in chronic myelogenous leukemia”.

#### Regulation relations

Regulation relations are similar to involvement relations except for the list of trigger words/phrases that can serve in the predicate position. Based on our previous work, the regulation trigger words we use include: regulates, promotes, induces, elevates, targets, enhances, increases, decreases, raises, up/down-regulates, modulates, causes, results, interacts, blocks, mediates, etc. In this category, we also encounter cases in which the predicate is expressed in a nominalized form. An example sentence is “restoration of mirna-143 (mir-143) regulates cox-2 and inhibits cell proliferation of pancreatic cancer cells”. Here the association between the miR-143 and the disease “pancreatic cancer” is via two linking entities: gene (cox-2) and cellular process (cell proliferation).

#### Association relations

To find sentences where a miR entity is associated with a process, linking entity, a disease or a disease outcome, we consider sentences with agent-predicate-theme dependencies with the following trigger words or phrases: “is associated with”, “correlated with”, “linked to”, etc. For example, in the sentence “reduced circulating mir-150 levels are associated with poor survival in pulmonary arterial hypertension.” a miR entity (expression of mir-150) is linked to a disease entity (“poor survival” in pulmonary arterial hypertension”).

#### Is-a relations

Relations that link two entities via the is_a relation are normally of agent-predicate-theme type. We expect the miR entity and the disease entity or linking entity to be picked as the agent and theme respectively, with “be” as trigger. However we include a wider range of trigger words or phrases such as:. is, are, acts as, functions as, serves as, etc. In the example sentence “Plasma miR-601 and miR-760 can potentially serve as promising non-invasive biomarkers for the early detection of colorectal cancer” both miR-601 and miR-760 are in a is_a relation with “non_invasive biomarkers” as indicated by the trigger phrase “serve as”. Note that sentence simplification makes it possible to link both miR to the theme “non-invasive biomarkers”.

#### Found_in

For detection of the “found_in” relation, we expect the use of the agent-predicate-theme dependency structure. Further, we expect the miR entity and the disease to be picked as the agent and theme respectively. These relations indicate that an aspect of the miR (expression, states like mutation, hyper-methylation) is found in the disease. There are two classes of triggers used to detect such relations. First set of triggers include words or multi-word triggers like: “is found in”, “is detected in”, “is increased in” etc. In these cases the miR entity will indicate the specific aspect of the miR (e.g. “*high level* of mir-155 was found in gallbladder cancer”). In other cases the aspect of the miR is inferred from the predicate trigger (e.g. “miR-155 was *overexpressed* in gallbladder cancer”). The triggers to detect such cases include: “overexpressed in”, “highly expressed in”, “upregulated in”, “mutated in” etc..

#### State relations

These relations are used to describe relations between a miR and its aspect or between disease and its aspect. In these sentences, the term describes the state in which the miR (or its promoter) is observed (e.g., overexpressed, methylated) or an outcome, treatment, or diagnostic property of the disease. Here we use the noun modification syntactic dependency, where miR aspect or disease aspect is the head noun and the miR or disease, respectively, is the modifier. For miR aspects we use trigger words such as methylation, expression, silencing, and knockdown while for disease aspects we use words/phrases such as level, biomarker, or disease free survival. An example sentence is “Overexpression of the miR-200b is associated with hepatocellular carcinoma cell migration through the epithelial to mesenchymal transition”.

##### Multiple predicate triggers

In some sentences there may be multiple predicate triggers appearing between an agent and a theme. For example in the sentence fragment “miR-9 is **involved** in the **regulation** of apoptosis…”, the two triggers “involved in” and “regulation” connect the miR entity (mir-9) and the linking entity (apoptosis). In such cases we can expect that one of the triggers will be semantically more specific. Typically, we have found that the more specific predicate is of the type “regulation” and the more general predicate is of the type “is-a” or “involvement”. Hence we modify the output to include only the more specific relation. For example we first extract the tuple [miR-9, is involved in, regulation of apoptosis] and resolve it to [miR-9, regulates, apoptosis], categorizing it as a “regulation” relation.

### Assigning scores to sentences

As we have seen from the above sections, there are many different ways in which a miR entity can be connected to a disease entity in a sentence through various linking entities and semantic relations. Biologists might be interested in sentences containing some categories of relation more than others, and might prefer sentences mentioning certain types of semantic relations more than others. We wanted to see if scoring sentences in terms of linking entities, semantic relations, and other factors (such as hedging), might help rank the sentences in a specific way that is preferred by biologists. Such a scoring system could potentially be used to prioritize the most relevant sentences when presenting miRiaD results to users.

Our assumption is that biologists might want to see sentences mentioning a miR-disease relationship that is explained via a target gene or a process. Equally important might also be sentences describing the miR-disease relationship via a sequence of biological steps connected by words such as “contributes”, “results in”, “causes”, “supports” and their textual variations, as well as the conjunction “and”. Less informative are sentences that describe the miR-disease relationship in terms of expression level of the miR. We assign a score between 1 and 3 based on how informative the sentence is (highly informative, informative, somewhat informative), using empirically–derived rules.

Sentences are assigned to the ***highly informative*** (3 points) category if they contain some explanation of the connection between the miR and the disease. We use two indicators for detecting the “explanatory” component. The first is the detection of null argument sentences. For example, in the sentence “**miR**-**137***functions as* a **tumor suppressor** by *targeting***CtBP1** to inhibit epithelial-mesenchymal transition and inducing apoptosis of melanoma cells” the target gene aspect of the miR explains its functionality as a tumor suppressor. The second indicator of the “explanatory” component is the presence of at least two semantic relations, which form a sequence of events/process/outcome/diagnostic/treatment.. One such example is “Treatment of gastric cells with dihydroartemisinin (DHA) *increased***miR**-**15b** and **miR**-**16 expression**, *caused* a *downregulation* of **Bcl**-**2**, *resulting in***apoptosis** of **gastric cancer** cells”. In addition we also assign sentences to the highly informative category if they contain target gene information together with some other relation (e.g., “UBASH3B is a functional target of anti-invasive miR200a that is down-regulated in triple negative breast cancer”).

Sentences are assigned to the ***informative*** (2 points) category if they contain both diagnostic and treatment disease aspects, a linking entity regulation process, or a treatment aspect of a disease in the theme (e.g., miR is a therapeutic target for a disease). Some example sentences in this category include: “miR-23b is epigenetically down-regulated and restoration of miR-23b can effectively suppress cell growth in glioma stem cells” or “miR-139-5p is a potential biomarker for early diagnosis and prognosis and is a therapeutic target for esophageal squamous cell carcinoma (ESCC)”.

Sentences are assigned as ***somewhat informative*** (1 point) if they contain an altered expression relation, an involvement relation, or a diagnostic aspect. Some examples include: “High miR-199a expression is associated with liver fibrosis” or “miR-125b could be an important prognostic indicator for colorectal cancer patients”. All sentences that were not assigned highly informative or informative scores are also considered to be somewhat informative.

A sentence can be upgraded from “somewhat informative” to “informative” or “informative” to “highly informative” if it contains an outcome aspect. A sentence can also be downgraded if it contains a diagnostic/treatment aspect that was obtained through a co-occurrence relation (i.e., not part of the relation itself but co-occurring in the sentence). Additionally, hedging or other evidence of doubt will lower the score of the sentence by one point. The triggers used here for doubt were “might”, “could”, “suggest”, “propose”, and their lexical variations.

### Evaluation: Overview and Experimental Setup

We evaluated miRiaD with respect to the needs of two potential user communities: miR-disease database curators and biomedical researchers studying disease mechanisms.

#### Study 1: Evaluation of miRiaD for assistance with manual curation

Database curators can benefit from automated text mining tools that make manual curation more efficient by quickly identifying relevant information in the literature. Because miR2Disease is the most comprehensive database aiming to manually curate miR-disease relationships, we focused our first study on the ability of miRiaD to retrieve the types of information annotated by miR2Disease, namely miR expression and target information in the context of disease. Initially, we tested the recall of miRiaD using sentences from abstracts that were cited as evidence in miR2Disease entries. By gathering the evaluation set from the database itself, we are guaranteed to have sentences that are of interest to miR2Disease curators; however, because all of the sentences are by definition positive, we cannot use them to assess precision. Therefore, we also evaluated the recall and precision miRiaD using a set of randomly selected Medline abstracts not found in the miR2Disease database. These abstracts were disease-focused and mentioned a miR, but only a subset had target gene or miR expression information, so we could test the ability of miRiaD to reject papers that are not relevant for curation by miR2Disease.

We downloaded the entire mirRDisease database, which contained 3273 entries at the time (January 2015). Each entry in the downloaded file contained a miR, a disease, a title of an article, and the year in which the article was published. Because no PMIDs were provided with the downloadable database, we looked up the PMID corresponding to each entry by matching the title and the year of publication in the Medline 2015 corpus. We filtered out the entries for which the miR and the disease mentions could not be found together in the abstract or the title of the article. This resulted in a total of 486 entries in the miR2Disease database that we could use for our study.

For these entries, we retrieved the title and the abstract of each referenced article from the Medline 2015 corpus. We also obtained the descriptions accompanying each miR-disease-article entry from the miR2Disease online database. These descriptions consist of sentences taken from the referenced article that support the miR-gene relation in the entry. Because we have so far only applied miRiaD to abstracts, we used Perl’s StringSimilarity Module, which is based on the algorithm described in [33], followed by manual verification to detect entries in which the description text was taken from the abstract. Of these entries, we randomly chose 100 to test the recall of the miRiaD system.

miR2Disease clearly mentions that their manual annotations include miRNA expression patterns in the disease state, detection methods for miRNA expression, and experimentally verified miRNA target gene(s). Therefore, we manually reviewed each description, and marked each sentence as containing expression and/or target information. Ninety descriptions were marked as containing expression information, and 58 descriptions were marked as containing target information.

Next, we selected randomly selected 100 additional Medline abstracts not found in the miR2Disease database. The abstracts were selected to be focused on diseases (i.e., a disease is mentioned in the title, the first or the last sentence of the abstract, or three or more times throughout the abstracts, as defined in the work by Tudor et al. [28]). One biologist annotator marked the abstracts in terms of genes targeted by the miR and miR expression information for the miR. There were 52 abstracts marked as containing expression information and 48 abstracts marked as containing target information.

#### Study 2: Evaluation for general extraction of miR-disease associations

For our second study, we evaluated miRiaD with respect to the extraction of a wide range of relations that appear in text connecting a miR to a disease that may be of interest to biomedical researchers. As above, we conducted the study in two parts: first, we selected a set which is highly likely to contain such relevant relation between a miR entity and a disease or linking entity and assessed recall and precision. Next we assessed precision and recall of relations for randomly selected sentences from Medline abstracts. For the first set, we gathered Gene Reference into Function (GeneRIF) sentences from the EntrezGene entries for miRs. GeneRIFs are used to annotate EntrezGene entries and consist of short sentences or phrases with literature citations describing the function of the gene/miRNA [34]. These GeneRIF sentences may or may not be direct quotes from the abstract and may be rephrased by the annotator. We chose these sentences because of their rich variety of miR-disease associations, which included relationships between miRs and miR aspects (expression) and diseases and disease aspects (outcome, biomarker, therapy), as well as linking entities (target, process). Importantly, for this study, we were not testing the ability of miRiaD to detect appropriate sentences for EntrezGene annotations; we were simply using the EntrezGene sentences as a convenient source of the types of relations we designed miRiaD to detect. The randomly chosen sentences mentioned both a miR and a disease but were both positive and negative for miR-disease associations. The annotators were asked to mark all the relations indicating a miR-disease association, such we can assess miRiaD’s ability to detect such relations.

We downloaded the GeneRIF sentences from the EntrezGene database (ftp://ftp.ncbi.nih.gov/gene/GeneRIF). The file contained a total of 946,742 entries at the time of download (January 2015). Each entry in the downloaded file contains a taxonomy ID, a gene ID, a PMID list, the GeneRIF text, and the timestamp of the last update. Using the gene ID field, we extracted all the entries where the gene ID was that of a miR. Additionally, we looked in the GeneRIF text of these entries for the mention of at least one disease using the PubTator database [20] for the detection of disease mentions. This resulted in a set of 8476 GeneRIF entries.

A set of 100 entries from all 8476 GeneRIF entries was randomly selected and presented to a second annotator. The annotator was asked to mark the GeneRIF sentences as relevant if they contained a relationship between a miR and a disease mentioned within or not relevant otherwise. Of the 100 sentences, 97 were marked as relevant and 3 were marked as not relevant. In addition, the annotator marked all of the relations in the sentence indicating an association between a miR and a disease, including miR and disease aspects and linking entities. Because multiple miRs, diseases, and types of relationships could be found within the same sentence, the annotations yielded a total of 175 relations.

We also randomly selected a second set of 100 sentences containing miR and disease mentions from Medline abstracts. As discussed earlier, the reason for selecting an additional 100 sentences was to construct a less biased evaluation set that included some negative sentences. Ninety-two sentences were marked as relevant and contained a total of 159 relations; 8 sentences were marked as not relevant.

#### Study 3: Evaluation of the miRiaD sentence informativeness ranking

Finally, we evaluated our informativeness ranking approach using a set of sentences containing miR and disease mentions that were manually scored for informativeness by a biologist. We selected 100 random sentences from the 8476 GeneRIF sentences that we obtained in the previous evaluation, plus 100 sentences randomly selected from Medline abstracts. We asked an annotator to mark each sentence with scores on a three-point Likert scale, depending on how “informative” the sentence might be to a researcher interested in the disease, with a score of 1 indicating “somewhat informative”, a score of 2 indicating “informative”, and a score of 3 indicating “highly informative”. The annotator marked 58 sentences as “highly informative”, 87 sentences as “informative”, and 55 sentences as “somewhat informative”.

## Results and Discussion

### Study 1: Evaluation of miRiaD for assistance with manual curation

miRiaD was applied to the sentences in 100 abstracts cited by miR2Disease and 100 randomly selected Medline abstracts (200 abstracts total) as described in Methods. The results of this evaluation are shown in Table 1. For the abstracts from miR2Disease, we obtained a recall of 90 % (81 true positives (TP) and 9 false negatives (FN)) for expression information and 89.6 % (52 TP and 6 FN) for target information. For the randomly selected abstracts, we got very similar results: recall of 92.3 % for expression information and 83.3 % for target information. When we combined the two sets of abstracts, we obtained recalls of 90.78 % and 88.46 % for expression and target information respectively. Using the randomly selected abstracts, we were able to assess precision as well as recall. For expression information we obtained precision of 92.3 % and f-score of 92.3 % (48 TP, 4 FN and 4 false positives (FP)); for target information we obtained precision of 97.5 % and f-score of 89.8 % for target information (40 TP, 8 FN and 1 FP).

**Table 1:** Evaluation of miRiaD for assistance with manual curation

	TP	FN	TN	FP	Recall	Precision	F-score
miR2Disease based set							
Expression	81	9	-	-	90.0	-	-
Gene target	52	6	-	-	89.6	-	-
Randomly chosen set							
Expression	48	4	46	4	92.3	92.3	92.3
Gene target	40	8	52	1	83.3	97.5	89.8
Combined							
Expression	129	13	-	-	90.78	-	-
Gene target	92	14	-	-	88.46	-	-

Looking at the false negatives, we observed that most of the errors were introduced by highly complex sentences, for which the iSimp tool could not generate useful simplified sentences. As a result, the syntactic and lexical patterns could not be matched. An example is the following sentence: “Among 15 upregulated target genes of the miR-30 miRNA, four genes known to be expressed and/or functional in podocytes were identified, including receptor for advanced glycation end product, vimentin, heat-shock protein 20, and immediate early response 3” (PMID 18776119). The four genes were not identified as targets of miR-30 due to the inability to link them to the “four genes” mention, and subsequently to the “15 upregulated target genes” mention. Another class of false negatives involves relations missed due to the presence of anaphora (generally a pronoun referring to an entity). For example in the sentence fragment “…miR-181a, a small non-coding RNA believed to induce apoptosis by repressing its target gene, BCL-2”, the word “its” refers to “miR-181a”. We currently do not perform pronoun resolution and hence miRiaD misses the target relation with “BCL-2”. Looking at false positives, we observed most of errors were introduced due incorrect identification of the simplification constructs. In the sentence “the protein inhibitor of activated STAT3 (PIAS3) was confirmed as a direct miR-21 target”, STAT3 was detected as the parenthetical for PIAS3 and tagged as a target for miR-21 in addition to PIAS3.

### Study 2: Evaluation for general extraction of miR-disease associations

For the second study, 100 GeneRIF sentences from EntrezGene entries for miRNA and 100 sentences with miR and disease mentions randomly chosen from Medline abstracts were processed by miRiaD (see Methods). The results of this evaluation are shown in Table 2. miRiaD was able to identify relevant relations in 91 sentences from the GeneRIF set and 93 sentences from the Medline set. For calculation of TP, FN and FP we matched the relations annotated by the annotators with the relations extracted by miRiaD. This yielded recall of 84.0 %, precision of 94.8 %, and f-score of 89.1 % with respect to GeneRIF sentences (147 TP, 28 FN and 8 FP) and recall of 84.2 %, precision of 96.4 %, and f-score of 89.8 % with respect to the additional Medline sentences (134 TP, 25 FN and 5 TP). As in the previous evaluation we do not see a significant difference in performance between the two evaluation sets. The combined f-score for all 200 sentences was 89.4 %.

**Table 2:** Evaluation for general extraction of miR-disease associations

	TP	FN	TN	FP	Recall	Precision	F-score
GeneRIF based Set	147	28	1	8	84.0	94.8	89.1
Randomly chosen set	134	25	7	5	84.2	96.4	89.8
Combined	281	53	8	13	84.1	95.5	89.4

Looking at the false negatives in this evaluation revealed an additional type of error. We observed certain syntactic dependencies between events that should be captured, but which are not. For example, the following sentence contains a temporal relation, which is triggered by the word “after”: “These data indicate for the first time a mechanism involving STAT1/2 upregulation under the transcriptional control of INF-alpha signaling after knockdown of miR-221/222 cluster in U251 glioma cells” (PMID 20428775). The relationship [miR-221/222, negatively regulates, STAT1] could not be extracted because of the system’s inability to detect the temporal relation. As in or previous evaluation, we observed false negatives due to anaphora. For example, in the sentence fragment “…the tumor suppressor activity of miR-124 could be partly due to **its** inhibitory effects on glioma stem-like traits and invasiveness…”, miRiaD misses the inhibitory relations between “mir-124” and “stem-like traits and invasiveness”.

Most of false positives errors in sentences had an involvement relation between a miR and a disease in addition to a more informative relation. For example in the sentence “this study further extends the biological role of miR-92b in non-small cell lung cancer A549 cells and for the first time identifies PTEN as a novel target of miR-92b”, miRiaD extracted an involvement relation between miR-92b and “non-small cell lung cancer” that was not marked as relevant by the annotator. Instead, the annotator only marked the relation between “mir-92b” and the target gene “PTEN” as relevant. This type of errors will constitute the grounds for future work.

### Study 3: Evaluation of the miRiaD sentence informativeness ranking

This evaluation was conducted on 100 sentences randomly selected from GeneRIF set used for the previous evaluation 100 sentences randomly selected from Medline abstracts, which were marked for their informativeness on a three-point scale by an annotator (see Methods). miRiaD ranked 57 sentences as “highly informative”, 97 sentences as “informative”, and 48 sentences as “somewhat informative”. The comparisons between the annotator’s scores and the miRiaD scores are shown in Table 3. Of the sentences ranked as “highly informative” by the system, most of them (45/57) were also ranked as “highly informative” by the annotator; the remaining 12 sentences were ranked as “informative” by the annotator. Looking at the diagonal of the table, we observe that the majority of the scores in all three categories were in agreement. The average score difference on the 3-point scale (absolute value) between annotator score and the miRiaD system score is 0.29.

**Table 3:** Evaluation of the miRiaD sentence informativeness ranking

Annotated	Highly informative	Informative	Somewhat informative	Total
System				
Highly informative	45	12	0	57
Informative	11	64	20	97
Somewhat informative	2	11	35	48
Total	58	87	55	200

Row values correspond to the frequency of scores assigned by miRiaD, while the columns denote the annotator score frequency

Various correlation measures were computed, where a value of 1 is total positive correlation, 0 is no correlation, and −1 is total negative correlation. First, we computed the Pearson product-moment correlation coefficient, which is widely used as a measure of the degree of linear dependence between two variables (0.727). Second, we computed the Kendall tau rank correlation coefficient, which measures the association between two measured quantities by looking at the similarity of the orderings of the data when ranked by each of the quantities (0.678). Finally, we computed the Spearman’s rank correlation coefficient, which assesses how well the relationship between two variables can be described using a monotonic function, and got a value of 0.724, which indicates a “very strong positive relationship” [35]. The *p*-values for these correlation scores are very low, indicating that the null hypothesis of no correlation is extremely unlikely. To test against a stronger baseline than “no correlation”, we randomly reordered the annotated scores 10,000 times and calculated the Pearson correlation coefficient. The mean correlation was 0.544, and none of the random correlation values were above the reported correlation value 0.727.

We also computed the normalized discounted cumulative gain (nDCG) [36, 37], which compares the ordering of a list by a system (e.g., miRiaD) with the perfect ordering of that list by gold standard (e.g., our annotator). Because the sentences were given scores between 1 and 3, and not actual ranks, multiple possible orderings are possible when displaying the sentences marked with 3 first, then the sentences marked with 2 second, and finally the sentences marked with 1 at the bottom. For this reason, we considered 10,000 different possible orderings. The mean of the nDCGs scores obtained this way was 0.977, with the lowest being 0.950 and the median being 0.978. The high nDCG values could be due to the strong agreement between miRiaD and the annotator for the sentences marked as “highly informative”.

### Browsing the results online

We have developed a preliminary website for interactive query of miRiaD miR-disease association extraction. The interface accepts PubMed-like queries as input, thus supporting queries like a miR name, or a disease name, or any biological concept. For example, a user interested in “miR-9” and “breast cancer” can submit a query such as “*mir*-*9*” *AND* “*breast cancer*”. To ensure that all of the abstracts passed to miRiaD contain a miR mention, we restrict the user query by appending the “AND” operator and miRNA keywords connected by the “OR” operator, i.e., *query AND* (*miRNA*[*TIAB*] *OR microRNA*[*TIAB*] *OR miR*[*TIAB*]). The system then submits the query to PubMed, which returns all the PMIDs satisfying the query. miRiaD processes this list of PMIDs and displays the triplets < miR,Disease,Text Evidence/PMID>. The interface is available at the URL: http://biotm.cis.udel.edu/miRiaD.

**Fig. 3:**
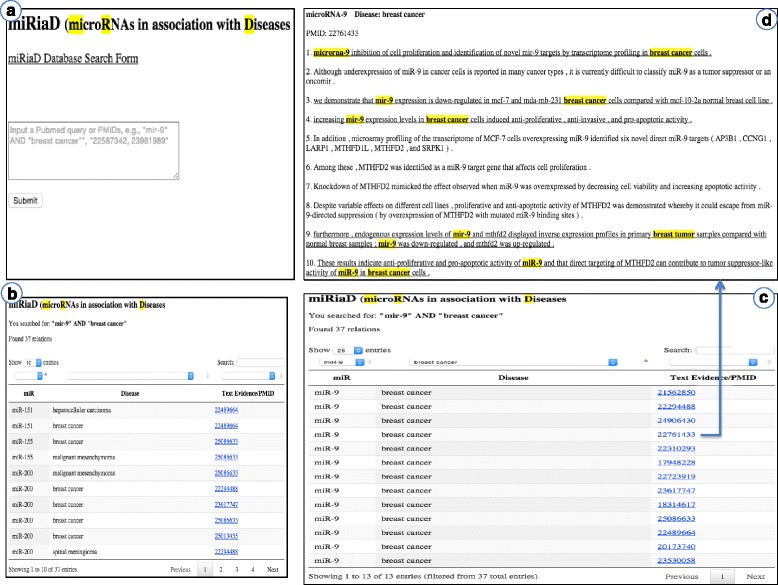
miRiaD Web Interface (http://biotm.cis.udel.edu/miRiaD). **a** Query interface. **b** Results of searching for “mir-9 AND breast cancer”. **c** Filtering of search results to show those where the miR is mir-9 and the disease is breast cancer. **d** Text evidence view with miR and disease mentions highlighted and miRiaD positive sentences underlined

Figure 3a–d provides screenshots of the interface. Figure 3a. shows the search form where the user submits his/her query. Figure 3b. shows the triplet view in the table after submitting the query “*mir*-*9*” *AND* “*breast cancer*”. There are three columns in the triplet view, namely: the miR, Disease and Text Evidence. Note that the results also contains triplets for other miR (mir-151, miR-200) and other diseases (hepatocellular carcinoma, Hodgkin’s lymphoma etc.). This is due to the fact that miRiaD processses the entire abstract for each PMID returned by the submitted query and some abstracts may contain mentions of multiple miR and/or diseases. Results can be filtered to include only those where mir-9 is the miR and breast cancer is the disease using the drop-down menus above each column as shown in Fig. 3c. Finally, clicking on the PMID in the Text Evidence column takes the user to the abstract (Fig. 3d.), where sentences indicating the miR-disease association are underlined and the respective miR’s and diseases are highlighted in bold.

Browsing the highlighted sentences in the 13 PMIDs shown in Fig. 3c reveals that miR-9 has been associated with a number of breast cancer phenotypes including metastasis, invasiveness, aggressiveness, cell motility, and poor prognosis. miR-9 targeting of E-cadherin (CDH1) has been implicated in promotion of cell motility and invasiveness. Variations in miR-9 expression related to miR-9 promoter hypermethylation have also been observed in the disease. As a consequence of these findings, miR-9 has been suggested as both a potential biomarker and therapeutic target. Interestingly, one study (PMID: 22761433) reported that miR-9 targeting of the mitochondrial enzyme, MTHFD2, mediated a tumor suppressive effect in breast cancer, and in contrast to other studies, found that miR-9 inhibited invasion. More careful consideration of the contextual information in these articles may help to resolve this apparent contradiction. This small example illustrates the wide variety of miRNA-disease information that can be easily obtained using miRiaD.

## Conclusion

In this paper, we have presented miRiaD, a relation extraction system that automatically extracts associations between miRs and diseases from the literature. The miR-disease associations are sometimes indirect, with miR and disease connected by a linking entity, such as a target gene or cellular process. Similarly, miRs and diseases might be mentioned in terms of their aspects: (e.g., state or expression level for miRs or outcomes, diagnostics/treatments, or therapy information for diseases). Our method, which entails detecting syntactic dependencies and, subsequently, semantic relations between miRs (or their aspects) and linking entities in the context of a disease or disease aspect, has demonstrated high performance (recall, precision, and F-scores) in identifying information of interest to both database curators and biomedical researchers studying the connections between miRs and disease. Additionally, we have shown how assigning scores to sentences containing a miR-disease association, based on syntactic dependencies, semantic relations, and linking entities, highly correlates with preferences that biologists have in selecting the most informative sentences for a miR-disease association. The evaluation studies also pointed out some pitfalls of the miRiaD system. Future work will include an expansion of the syntactic dependencies, in conjunction with the iSimp sentence simplifier, to detect multi-step causal events starting from a miR and leading to its involvement in a disease.

We have provided an online interface for browsing the results extracted from the entire Medline corpus, searchable using PubMed queries. Our next steps will be focused on full-scale processing of the PubMed Central (PMC) Open-Access collection and enhancement of the miRiaD on-line interface to allow for more complex querying of the results. This work will enable researchers to address sophisticated questions concerning the pathological roles of miRs, such as (i) Are there miRs associated with metastasis across multiple cancer types? (ii) Is there overlap in the sets of genes targeted by miRs in different types of dementia? (iii) Which miRs are associated with a better prognosis in prostate cancer? (iv) For miRs that have been identified as possible therapeutic targets in breast cancer, what biological processes do they regulate in a breast cancer context? (v) In what diseases does miR regulation of apoptosis play a role? We plan to conduct specific biological use-case studies aimed at some of these questions in the near future.

## Additional file

Additional file 1:
**Formal description of the syntactic dependencies patterns.** A formal description of the patterns used in detection of syntactic dependencies is provided in this file. In addition the list of triggers used for determining semantic relation type and the types semantic relations between the different entities is also provided in this file. (PDF 83 kb)
