# Supplementary material for: miRiaD: A Text Mining Tool for Detecting Associations of microRNAs with Diseases
Source: J Biomed Semantics. 2016 Apr 29;7:9. doi: 10.1186/s13326-015-0044-y (PMC4877743; doi:10.1186/s13326-015-0044-y)
Supplement: Additional file 1: — Formal description of the syntactic dependencies patterns. A formal description of the patterns used in detection of syntactic dependencies is provided in this file. In addition the list of triggers used for determining semantic relation type and the types semantic relations between the different entities is also provided in this file. (PDF 83 kb) [file 13326_2015_44_MOESM1_ESM.pdf]

Syntactic Dependencies Patterns:

1. Agent-Theme of Predicate

In all cases below "arg1" refers to the agent of the predicate and "arg2" the theme of the predicate. In all these case arg1/arg2 is either a base Noun Phrase (NP) or NP with prepositional attachments. VG+ is a verb group or multiple verb groups.

1a. Active form:

Pattern #1: <arg1> VG+ <arg2>

To detect the predicate trigger word we have three different cases:

(i) VG+ is a single verb group (e.g. "regulates", "directly regulates" etc.). In these cases the predicate trigger is the head of the Verb Group (rightmost verb of the Verb Group).

(ii) VG+ is a sequence of two verb groups (e.g. "is known to regulate", "found to is found to induce" etc.). In the VG+ "is known to regulate" the first VG is "is known" and the second VG is "to regulates". In these cases the rightmost VG has an active form and other VG can be in passive form. Here the predicate trigger is the head of the second VG.

Pattern #2: <arg1> VG+ NP prep <arg2>

Here the phrase connecting arg1 and arg2 will be a VG+ followed by a base noun phrase followed by a preposition (e.g. "plays a role in", "is essential for" etc.). In the phrase "plays a role in" the VG+ is "plays", NP is "a role" and the prep is "in". In these case the predicate trigger is the head of the NP (rightmost noun).

1b. Passive form

Pattern: <arg2> VG+ "by" <arg1>

In these case the VG+ will start with a "be" verb (is, are , was , were etc.) and the head verb (rightmost verb) of the Verb Group will be in the past participle form (e.g. "is regulated by", "is mediated by").

1c. Nominalized form

These cases involve where the predicate trigger is in the nominalized form of a verb (e.g. "regulation", "mediation", "induction" etc.).

Pattern #1: NP "of" <arg2> by <arg1>
Example: Regulation of apoptosis by mir-9

Pattern #2: NP of <arg2>
Example: "mir-9 regulation of apoptosis"

Pattern #3: NP by <arg1>
Example "smad-2 regulation by mir-145"

The predicate trigger is the head noun of the NP. Notice in Pattern#2 arg1 (mir-9) is part of the NP containing the nominalized form of the verb. Also in Pattern#3 arg2 (smad-2) is part of the NP containing the nominalized form of the verb. The arguments (arg1 or arg2) are modifier (left words) of the nominalized form of the verb. Notice the nominalized adjectives attached to it ("direct regulation", "epigenetic activation" etc.).

1d. Null-argument Case

Pattern #1 : NP1 VG1+ NP2 "to" VG2+ NP3
Other Constraint: The head verb of VG2+ will be a simple verb ("regulate", "induce" etc.)
Example: mir-145 targets smad2 to regulate cell-proliferation.

Pattern #2 : NP1 VG1+ NP2 "by" VG2+ NP3
Other Constraint: The head verb of VG2+ will be a verb in "ing" form ("regulating", "inducing" etc.)
Example: mir-145 regulates cell proliferation by targeting smad-2

Pattern #2 : NP1 VG1+ NP2 "via/through" VG2+ NP3
Other Constraint: The head verb of VG2+ will be a verb in nominalized form ("regulation", "induction" etc.)
Example: mir-145 regulates cell proliferation via regulation of smad2.

From all of the above patterns, we extract two syntactic dependencies

1. NP1 VG1+ NP2
2. NP1 VG2+ NP2

The arguments (arg1 and arg2) and predicate triggers are then detected with one of the case 1a-c.

2. Noun Modification

In these cases we detect the following syntactic dependency (<NG1> mod <NG2), where NG is a noun group. Here NG1 is the "modifier" and NG2 is the "head"

Pattern #1: NP
Here the first word/phrase of NP is NG1 and head word/phrase of NP is NG2. Notice NG could be multiple words based on named entity detection (miR, disease, outcome, expression). Thus is the sentence fragment "colon cancer metastasis", "colon cancer" is NG1 and not "colon" because "colon cancer" is named entity. Thus the syntactic dependency extracted in this case is ("colon cancer", mod, "metastasis")

Pattern #2: NP2 "of/for" NP1
In this case NP2 is NG2 and NP1 is NG1. (e.g. metastasis of colon cancer)

Semantic Relations

Based on the predicate trigger we assign semantic category to the relation. The triggers are listed below for each semantic type. Example trigger words are given below for each category. Although only some forms of trigger words are provided, the reader should assume all of their textual variations (tense and nominalized forms).

1. Involvement

This relation follows the agent-theme of predicate relation. The agent can be miR entity and the theme a disease entity or a linking aspect. The triggers used to detect involvement relations are: is involved in, is implicated in, is required for, is needed for, play a role in, is necessary for, is dependent on, participates in, contributes to, influences, fosters, affects, allows, initiates, important for, needed for, sufficient for, needed for, sufficient for, required for, critical for, crucial for, essential for, influences, fosters, effect in, allow, initiates.

2. Regulation

This relation follows the agent-theme of predicate relation. The agent can be miR entity and the theme a disease entity or a linking aspect. The triggers used to detect regulation relations are : regulates, promotes, induces, elevates, targets, enhances, increases, decreases, raises, up/down-regulates, modulates, causes, results, interacts, blocks,

mediates, accelerates, attenuates, diminish, decrease, downregulate, upregulate, reduce, abolish, abrogate, annul, block, activate, delete, disrupt, eliminate, impair, inhibit, interfere, knockdown, limit, prevent, repress, restrict, suppress, change, stimulate, stabilize, transform, mediate, prolong, result in, yield, enhance.

3. Association

This relation follows the agent-theme of predicate relation. The agent can be miR entity and the theme a disease entity or a linking aspect. As the association relation is symmetrical, the agent theme roles can be switched and thus a disease/linking entity can be the agent and the miR entity the theme. The triggers used to detect association relations are: is associated with, correlated with, linked to, contemporaneous with.

4. IS-A

This relation follows the agent-theme of predicate relation. The agent can be miR entity and the theme a disease entity. The triggers used to detect is-a relations are: is, are, acts as, functions as, serves as, encodes. Any multi word trigger ending with “as” or “be” are also used to detect is-a relations.

5. Found_in

This relation follows the agent-theme of predicate relation. The agent can be miR entity and the theme a disease. The triggers used to detect found_in relations are: found in, detected in, changed in, observed in, discovered in, over/under expressed in, up/down regulated in, methylated in, decreased in, increased in, silenced in, reduced in, hyper/de methylated in, dysregulated in.

6.State

This relation follows the noun-modification relation. The head is a miR aspect (expression, states like “mutation”, hypermethylation” etc.) and the modifier a miR mention (e.g. miR-9). The head can also be disease aspect (biomarker, treatment, diagnostic) and the corresponding head the disease mention. The triggers to detect this relation depend on the triggers used to detect miR/disease aspect, which are listed and discussed in the manuscript.
